# Illness perceptions in patients receiving rheumatology rehabilitation: association with health and outcomes at 12 months

**DOI:** 10.1186/1471-2474-14-28

**Published:** 2013-01-16

**Authors:** Ida Løchting, Elin Fjerstad, Andrew M Garratt

**Affiliations:** 1 National Resource Centre for Rehabilitation in Rheumatology (NRRK), Department of Rheumatology, Diakonhjemmet Hospital, P.O. Box 23 Vinderen, Oslo, 0319, Norway; 2 Communication- and Research Unit for Musculoskeletal Disorders (FORMI), Oslo University Hospital, Ullevaal, P.O. Box 4950 Nydalen, Oslo, 0424, Norway; 3 Norwegian Knowledge Centre for the Health Services, P.O. Box 7004, St Olavs plass, Oslo, N-0130, Norway

**Keywords:** Rehabilitation, Rheumatic diseases, Illness perceptions

## Abstract

**Background:**

Illness perceptions have been found to change over time and following health care. Hence, addressing illness perceptions alongside existing health care interventions may be important for the sustainment of health gains following rehabilitation. The aim of this study was to measure the illness perceptions of patients receiving inpatient rheumatology rehabilitation and assess the association with aspects of health and outcomes at baseline, discharge and 12 months.

**Methods:**

Patients with a rehabilitation stay of one week or more at three institutions in Norway in 2009 were invited to participate in the study. At baseline, discharge and 12 months, patients completed The Rheumatic Disease Illness Perception Questionnaire (RD-IPQ) which includes aspects of illness perceptions important to patients with rheumatic diseases. Stepwise regression analysis was used to assess associations between RD-IPQ scores and different aspects of health at baseline and follow-up after controlling for other aspects of health and sociodemographic variables.

**Results:**

For the 134 patients included in the study, baseline RD-IPQ scores had a mean of 58.2 (SD 14.9) on a 0–100 scale, where 100 is the worst possible. Scores showed improvement after the rehabilitation stay which were maintained at 12 months. RD-IPQ scores were positively associated with health and outcomes. At baseline RD-IPQ scores were statistically significant in explaining variation in pain, physical function and SF-36 mental health scores. Baseline RD-IPQ scores were significant in explaining fatigue, pain, SF-36 role limitations and social function scores following rehabilitation and at 12 months.

**Conclusion:**

Illness perceptions as measured by the RD-IPQ were associated with health and outcomes as measured by rheumatology-specific and generic instruments. The consideration of illness perceptions as a component of rehabilitation may be important in achieving desired outcomes.

## Background

The World Health Organisation (WHO) classification has over 200 musculoskeletal diseases or conditions [1]. Among the most prevalent are rheumatoid arthritis and osteoarthritis. Musculoskeletal disease affects joints, bones, soft tissues and muscles with important implications for health and quality of life. Many patients require some form of rehabilitation throughout their life and musculoskeletal disease accounts for the largest proportion of health and social benefits payments in Norway [2].

Rheumatology rehabilitation often includes a multidisciplinary team approach [3] and the biopsychosocial model has received increased attention in recent years [4,5]. This model proposes that experiences of illness and symptoms are influenced by biological, psychological and social factors. Dysfunction is not only related to the severity of disease, but also to how illness is perceived. The patient’s beliefs and perceptions relating to their illness are associated with how the illness affects them both physically and emotionally. Illness perceptions are not only based on symptoms, but also on the illness related consequences, associated anxiety and past experiences of illness [6].

Recent research has found that illness perceptions are important in explaining variations in outcomes in chronic illness [6] including rheumatic disease [6-8]. This research is mostly guided by a model of illness representations which postulates that patients’ views about their illness are based around five interrelated components; beliefs about consequences, beliefs about control and cure, causal beliefs, identity of their illness, and timeline [9]. In addition to these cognitive perceptions, patients’ have emotional responses to illness including anger, anxiety and depression. Each of these components relates to a perception about one aspect of the illness and together they provide the patient’s coherent view of an illness. Illness perceptions influence how the patient adjusts and copes with their illness [9].

In a recent UK study of over 2000 patients with osteoarthritis, it was found that patients with a strong illness identity, whose illness had a negative effect on their lives, were more likely to experience reduced function, greater pain levels, greater use of medication and more GP consultations [7]. Research in primary care has found that the way patients cognitively try to make sense of their illness affects self-perceived physical and mental health over time [7,10,11]. In addition, beliefs relating to more severe disease consequences have been found to be associated with higher levels of work-related disability for patients with chronic illness [12]. Studies have found that illness perceptions change over time and following health care [11,13,14]. Hence, understanding and addressing illness perceptions alongside existing health care interventions may be important for achieving desired health outcomes [15] and for the sustainment of health gains following rehabilitation.

This study aims to measure the illness perceptions of patients with rheumatic disease having an inpatient rehabilitation stay and to assess the association between these perceptions and health and outcomes after controlling for sociodemographic variables at baseline, discharge and after 12 months.

## Methods

### Data collection

Data collection took place over a nine month period from January to September 2009 and 208 patients scheduled for an inpatient rehabilitation stay of one week or more in one of three rehabilitation institutions in the South-East of Norway, were invited to take part in the study. Exclusion criteria were age over 75 years, unable to read and write Norwegian and cognitive dysfunction. Data collection procedures meant that information relating to the total number of patients asked to participate was unobtainable. All patients participating in the study gave written consent according to the Declaration of Helsinki. The study was approved by the Norwegian Regional Committee for Medical Research Ethics and the Data Inspectorate.

### Patient- reported outcomes

Patients completed a questionnaire that included questions relating to illness perceptions, health, outcomes and sociodemographic characteristics on arrival, discharge and after 12 months.

The 11-item Rheumatic Disease Illness Perception Questionniare (RD-IPQ) adapted from the original IPQ for patients with rheumatic diseases comprises cognitive and emotional illness perceptions including illness cause, comprehension, consequences, emotions, fluctuations, identity, personal control and treatment control [16] . The questionnaire asks about illness perceptions in the last two weeks and items have a five-point descriptive scale from “not at all” to “to a very large extent”. An overall scale of illness perceptions is computed by summing the items representing illness concequences, illness emotions, illness identity. The instrument has acceptable evidence for data quality, reliability, validity and responsiveness in rheumatology rehabilitation. An English version of the RD-IPQ is available at the National resource centre for rehabilitation in rheumatology’s (NRRK) website; (http://www.diakonsyk.no/modules/module_123/proxy.asp?D=2&C=634&I=8061).

Physical function was assessed by the 8-item Modified Health Assessment Questionnaire (MHAQ) [17,18]. MHAQ items have a four-point scale from “without any effort” to “not capable” and sum to give an overall mean score from 0 to 3 where 3 is the greatest overall disability. General health were assessed by the Short -Form 36- item (SF-36) health survey which comprises 36 questions with between three- and six-point scales that form eight scales with scores from 0 to100 where 100 is the best possible health [19,20]. Patients also completed two questions relating to fatigue and pain in the last week on a ten-point numerical rating scale (NRS) [21,22] where 10 is the worst possible fatigue or pain. The questionnaire also included questions relating to diagnosis, age, gender and education.

### Statistical analysis

The paired sample t-test was used to evaluate changes in illness perceptions over time. The contribution of RD-IPQ scores to baseline health and health outcomes after discharge and twelve months was assessed through forward stepwise regression analysis [23] after controlling for potential confounders.

The dependent variables included three widely used instruments within rheumatology, MHAQ and NRS fatigue and pain, and the SF-36 scales of social functioning, role limitations and mental health; aspects of health not covered by MHAQ and NRS. The exclusion of the scales that assess the same aspects of health was designed to circumvent the problem of multicollinearity and preference was given to instruments more widely used within rheumatology. The scores for the remainder instruments were included as potential independent variables. Possible confounders included primary rheumatic diagnosis, number of years since diagnosis, age, gender, education level (higher education or not), receipt of disability allowance, sick leave and old age pension.

The follow-up scores for the MHAQ, NRS and four SF-36 scales were the dependent variables in further sets of analyses. The baseline scores for these instruments were also included as potential independent variables together with the same disease-related and sociodemographic variables.

The 5% significance level was used for the multivariate analysis. It is widely recognized that stepwise regression may not give the best model if there is a high levels of correlation between independent variables. Multicollinearity was assessed using tolerance estimated as 1 – R^2^ which should be at least 0.10 [23,24] but higher values of 0.20 have been recommended [25]. SPSS version 17.0 was used for statistical analysis.

## Results

### Data collection

The questionnaire was returned by 134 patients. Their mean age was 55.38 (SD 10.23) years, the majority were female (86.6%) and reported having one rheumatic disease (64.8%) (Table 1).

**Table 1:** Baseline patient characteristics (n= 134)

	**N (%)**
Diagnosis*	
Inflammatory disease^a^	49 (36.5)
Osteoarthritis	25 (18.7)
Fibromyalgia	11 (8.2)
Polymyalgia rheumatica	1 (0.7)
Other rheumatic disease	1 (0.7)
More than one rheumatic disease	46 (34.3)
Sex	
Male	18 (13.4)
Females	116 (86.6)
Age mean (sd)	55.38 (10.2)
Education	
9 years	36 (26.9)
12 years	43 (32.1)
> 12 years	55 (41.0)
Current work status	
Employed	28 (20.9)
Not employed/sick leave	106 (79.1)
RD-IPQ mean (sd) ^b^	58.22 (14.93)
MHAQ mean (sd) ^c^	0.5 (0.3)
NRS mean (sd) ^d^	
Pain	5.5 (2.0)
Fatigue	6.5 (2.3)

*The diagnosis does not add up to 100% because one patient had missing data.

^a^ Rheumatoid arthritis, connective tissue disease, spondyloarthropathies.

^b^ RD-IPQ is scored from 0 to 100 where 100 is worst possible illness perceptions.

^c^ MHAQ is scored from 0–3; 3 is the greatest overall disability.

^d^ NRS scales are scored from 0–10; 0 is no pain and fatigue,10 is the worst pain and fatigue.

There were no differences in respondents and non-respondents at the discharge (n=114) and at 12 months (n=93) follow-up in relation to NRS pain, NRS disease activity, diagnosis, age, gender, education and work status.

Mean RD-IPQ scores were 58.35 (SD 14.99), 52.34 (SD 15.41) and 54.14 (SD 14.44) at baseline, discharge and 12 months respectively (Table 2). The score improvements were statistically significant at both discharge and at 12 months. The poorest scores were found for the identity items. The treatment control and comprehension items had the best mean scores. Significant improvements from baseline to discharge and from baseline and 12 months were found for items measuring identity, consequences, personal control and comprehension (Table 2).

**Table 2:** Mean (SD) for RD-IPQ scores at baseline, discharge and 12 months

**RD-IPQ **^**a**^	**Baseline (n=112)**	**Discharge**	**Baseline (n=93)**	**12 months**
*RD-IPQ sumscale *^b^	58.35 (14.99)	52.34 (15.41)***	58.22 (13.69)	54.14 (14.44)***
Experienced symptoms (identity)	2.73 (0.67)	2.46 (0.76)***	2.76 (0.62)	2.63 (0.69)
Symptoms affected your life (identity)	2.71 (0.78)	2.28 (0.73)***	2.74 (0.74)	2.44 (0.71)***
Negative effect on your life (concequences)	2.46 (0.75)	2.24 (0.83)**	2.45 (0.75)	2.29 (0.77)*
Good life in spite of disease (concequences)	1.75 (0.74)	1.54 (0.71)**	1.75 (0.74)	1.52 (0.78)**
Worried (emotions)	2.17 (0.91)	2.05 (0.92)	2.09 (0.83)	2.09 (0.76)
Negative emotions (emotions)	2.18 (1.01)	1.97 (0.95)	2.14 (0.93)	2.02 (1.01)
Able to influence disease (personal control)	2.17 (0.68)	2.02 (0.66)*	2.20 (0.68)	1.99 (0.64)**
Clear understanding of disease (comprehension)	1.71 (0.86)	1.50 (0.76)**	1.70 (0.90)	1.40 (0.78)*
Thought health care can help (treatment control)	1.44 (0.86)	1.50 (0.82)	1.44 (0.83)	1.51 (0.80)
Experienced fluctuations in disease (cyclical)	2.25 (0.78)	2.25 (0.76)	2.25 (0.77)	2.16 (0.77)

^a^ Items are scored on a 5-point scale from 0–4 (not at all - to a very large extent). Item 4,7,8 and 9 in this table are revised prior to analysis.

^b^ The 6-item RD-IPQ scale is scored from 0–100; 0 and 100 are the best and worst possible illness perceptions.

Asterisks denote statistical significance: *p<0.05; **p<0.01 level ***p<0.001 Paired sample t-test.

Table 3 shows the results of the stepwise multiple regressions analyses at baseline with the MHAQ, NRS and SF-36 scales as dependent variables. Between three and seven variables explained statistically significant components of the variation and entered the seven equations. Baseline RD-IPQ scores had a statistically significant association with three of the dependent health-related variables after controlling for other aspects of health status, years since diagnosis, diagnosis and sociodemographic variables. Patients with more positive illness perceptions have higher levels of health status as measured by the MHAQ, NRS pain and SF-36 mental health scales. For example, the RD-IPQ was the most important explanatory variable when the MHAQ scores were the dependent variable with the SF-36 scale of role-physical and male gender also entering the equation.

**Table 3:** **Stepwise regression**^**a **^**of baseline scores for the MHAQ, NRS fatigue, NRS pain and SF-36 on baseline scores for the RD-IPQ and other variables (n= 118)**

**Dependent vbl**	**Independent vbls**	**Regression coefficient**	**Standard error**	**t**	**Significance level**	**R2**^**b**^
MHAQ^c^	RD-IPQ^d^	0.007	0.002	3.73	.000	0.241
	SF-36 role physical	−0.005	0.001	−4.12	.000	0.318
	Male	0.207	0.078	2.63	.010	0.357
	(Constant)	0.253	0.154	1.64	.104	
NRS ^e^ fatigue	SF-36 social function	−0.030	0.008	−3.88	.000	0.175
	NRS pain	0.439	0.102	4.28	.000	0.252
	Inflamm disease	1.249	0.386	3.23	.002	0.315
	(Constant)	5.464	0.898	6.09	.000	
NRS pain	SF-36 role-physical	−0.027	0.007	−4.22	.000	0.325
	RD-IPQ	0.052	0.012	4.44	.000	0.435
	NRS fatigue	0.202	0.061	3.29	.001	0.463
	SF-36 mental health	0.022	0.008	2.62	.010	0.486
	Inflamm disease	−0.733	0.283	−2.59	.011	0.515
	(Constant)	1.199	1.222	0.98	.329	
SF-36^f^ role-physical	NRS pain	−3.983	0.862	−4.62	.000	0.325
	MHAQ	−18.928	4.807	−3.94	.000	0.410
	SF-36 social function	0.214	0.073	2.92	.004	0.471
	Male	13.968	4.546	3.07	.003	0.496
	SF-36 role-emotional	0.228	0.067	3.39	.001	0.521
	Higher education	−7.430	3.017	−2.46	.015	0.544
	SF-36 mental health	−0.233	0.113	−2.06	.042	0.561
	(Constant)	65.055	8.69	7.49	.001	
SF-36 social function	SF-36 mental health	0.593	0.098	6.06	.000	0.305
	SF-36 role-physical	0.312	0.078	3.98	.000	0.416
	NRS fatigue	−2.541	0.768	−3.31	.001	0.466
	Age	0.385	0.172	2.25	.027	0.489
	(Constant)	0.525	13.858	0.04	.970	
SF-36 role-emotional	SF-36 mental health	0.846	0.111	7. 60	.000	0.389
	SF-36 role-physical	0.393	0.088	4.48	.000	0.478
	No. yrs diagnosed	−0.545	0.261	−2.09	.039	0.497
	(Constant)	−1.24	8.493	−0.15	.885	
SF-36 mental health	SF-36 role-emotional	0.260	0.046	5.63	.000	0.389
	SF-36 social function	0.216	0.054	3.99	.000	0.490
	RD-IPQ	−0.389	0.108	−3.62	.000	0.516
	NRS pain	2.132	0.719	2.96	.004	0.551
	(Constant)	48.990	8.773	5.58	.000	

^a^ Variables are listed in the order in which they entered the equations and the results.

^b^ Proportion of variation that these variables account for in dependent variable scores.

^c^ MHAQ is scored from 0–3; 3 is the greatest overall disability.

^d^ RD-IPQ is scored from 0 to 100 where 100 is worst possible illness perceptions.

^e^ NRS scales are scored from 0–10; 0 is no pain and fatigue,10 is the worst pain and fatigue.

^f^ SF-36 scales are scored from 0 to100 where 100 is the best possible health.

Table 4 shows the results with the discharge scores for the MHAQ, NRS and SF-36 as dependent variables. The results are not shown for the MHAQ and NRS pain as dependent variables because only their respective baseline scores were significant explanatory variables. Up to three variables explained statistically significant components of the variation in the remainder of the scores. For example, when the NRS fatigue scores were the dependent variable, the baseline scores for both the NRS fatigue and RD-IPQ were significant explanatory variables. Baseline RD-IPQ scores also had a statistically significant association with follow-up scores for the SF-36 scales of role-physical, social function and role-emotional. This shows that patients with better levels of baseline illness perceptions have more positive health outcomes at discharge as assessed by these four variables.

**Table 4:** **Stepwise regression**^**a **^**of discharge scores for NRS fatigue and SF-36 on baseline scores for the RD-IPQ and other variables (n= 97)**

**Dependent vbl - discharge**	**Independent vbls - baseline**	**Regression coefficient**	**Standard error**	**t**	**Significance level**	**R2**^**b**^
NRS^c^ fatigue	NRS fatigue	0.427	0.104	4.10	.000	0.200
	RD-IPQ ^d^	0.034	0.017	2.02	.046	0.234
	(Constant)	1.298	1.032	1.26	.212	
SF-36^e^ role-physical	SF-36 role-physical	0.414	0.098	4.23	.000	0.294
	Inflamm disease	12.499	4.454	2.81	.006	0.359
	RD-IPQ	−0.323	0.157	−2.06	.042	0.387
	(Constant)	46.738	12.038	3.88	.000	
SF-36 social function	RD-IPQ	−0.478	0.182	−2.63	.010	0.203
	SF-36 mental health	0.380	0.153	2.49	.015	0.252
	(Constant)	68.303	19.048	3.59	.000	
SF-36 role-emotional	SF-36 role-emotional	0.338	0.092	3.68	.000	0.284
	RD-IPQ	−0.500	0.177	−2.82	.000	0.335
	No. yrs diagnosed	−0.954	0.353	−2.70	.008	0.383
	(Constant)	85.571	15.292	5.60	.000	
SF-36 mental health	SF-36 mental health	0.787	0.082	9.58	.000	0.481
	MHAQ ^f^	−15.465	4.822	−3.21	.002	0.509
	SF-36 role-physical	−0.164	0.069	−2.36	.020	0.537
	(Constant)	30.282	7.118	4.25	.000	

^a^ Variables are listed in the order in which they entered the equations. The results for the analyses with the MHAQ and NRS pain as dependent variables are not shown because only the baseline scores for the same instruments entered the equations.

^b^ Proportion of variation these variables are able to account for in dependent variable scores.

^c^ NRS scales are scored from 0–10; 0 is no pain and fatigue,10 is the worst pain and fatigue.

^d^ RD-IPQ is scored from 0 to 100 where 100 is worst possible illness perceptions.

^e^ SF-36 is scored from 0 to100 where 100 is the best possible health.

^f^ MHAQ is scored from 0–3; 3 is the greatest overall disability.

Table 5 shows the results with the 12 months scores for the same instruments as dependent variables. The results are not shown for the MHAQ because only the MHAQ baseline scores were a significant explanatory variable. Up to three variables explained statistically significant components of variation in the remainder of the scores. For example, when the NRS pain scores were the dependent variable, both the NRS pain and RD-IPQ baseline scores were significant explanatory variables. RD-IPQ baseline scores were also significantly associated with 12 month scores for SF-36 role-physical and social function scales (Table 5). Patients with better illness perceptions at baseline have less pain and report better functioning as measured by SF-36 role-physical and social function scales at 12 months.

**Table 5:** **Stepwise regression **^**a **^**of 12 month scores for the MHAQ, NRS fatigue, NRS pain and SF-36 on baseline scores for the RD-IPQ and other variables (n=80)**

**Dependent vbl - discharge**	**Independent vbls - baseline**	**Regression coefficient**	**Standard error**	**t**	**Significance level**	**R2**^**b**^
NRS ^c^ pain	NRS pain	0.402	0.008	3.79	.000	0.270
	RD-IPQ ^d^	0.035	0.106	2.36	.021	0.319
	(Constant)	1.094	0.015	1.34	.184	
NRS fatigue	NRS fatigue	0.641	0.099	6.46	.000	0.355
	Male	−1.977	0.795	−2.49	.015	0.403
	(Constant)	2.416	0.728	3.32	.001	
SF-36^e^ mental health	SF-36 mental health	0.921	0.094	9.83	.000	0.559
	SF-36 role-emotional	−0.148	0.058	−2.55	.013	0.593
	(Constant)	16.907	5.340	3.17	.002	
SF-36 role-emotional	SF-36 mental health	0.740	0.134	5.53	.000	0.251
	Pensioner	−23.377	7.274	−3.21	.002	0.338
	(Constant)	26.560	9.518	2.79	.007	
SF-36 role-physical	NRS pain	−4.171	1.448	−2.88	.005	0.200
	Osteo	15.527	6.297	2.47	.016	0.269
	RD-IPQ	−0.509	0.207	−2.46	.016	0.322
	(Constant)	98.418	11.487	8.57	.000	
SF-36 social function	SF-36 social function	0.379	17.186	3.19	.002	0.216
	RD-IPQ	−0.545	0.119	−2.50	.014	0.274
	(Constant)	65.650	0.218	3.82	.000	

^a^ Variables are listed in the order in which they entered the equations. The results for the analyses with the MHAQ as dependent variable are not shown because only the baseline scores for the MHAQ entered the equation.

^b^ Proportion of variation these variables are able to account for in dependent variable scores.

^c^ NRS scales are scored from 0–10; 0 is no pain and fatigue,10 is the worst pain and fatigue.

^d^ RD-IPQ is scored from 0 to 100 where 100 is worst possible illness perceptions.

^e^ SF-36 is scored from 0 to100 where 100 is the best possible health.

The minimum tolerance found for any variable in the regression analyses was 0.43 which meets the criterion of 0.20 [24]. Moreover, the great majority were in excess of 0.60 which is evidence that multicollinearity has not adversely affected the results.

## Discussion

This study assessed the illness perceptions of patients attending rheumatology rehabilitation at baseline, discharge and 12 months. At baseline patients had a relatively strong illness identity, consequences and negative emotions with mean RD-IPQ scores of 58.2 (SD 14.9). Significant improvements in both item and RD-IPQ scale scores were seen shortly after the rehabilitation stay and at 12 months.

The results of multiple regression with the MHAQ, NRS and SF-36 as dependent variables at baseline shows that having a strong illness identity, experiencing more illness consequences and more negative emotions relating to the illness are associated with greater disability, worse pain and poorer mental health. This follows previous findings from studies that have found an association between illness perceptions and outcome [6]. One study of patients with fibromyalgia attending rehabilitation showed that the same three aspects of the RD-IPQ scale scores were those found to be associated with health at rehabilitation baseline [26].

Furthermore, the current study found that baseline levels of illness perceptions were associated with outcomes at discharge and 12 months. Better levels of illness perceptions at baseline as assessed by the RD-IPQ were associated with greater health on the NRS fatigue and SF-36 scales of role-physical, social function and role-emotional scale at discharge and on the NRS pain and SF-36 scales of role-physical and social function at 12 months. Furthermore, for both discharge and 12 month outcomes, none of the other potential explanatory variables entered the equations as often as the RD-IPQ scores which show that illness perceptions may make a relatively important contribution to health outcomes.

Studies relating to rheumatology rehabilitation have shown that patients improve after a rehabilitation stay, but that statistically significant improvement often is lost at follow up [27,28]. The current study found that illness perceptions improve and are associated with outcomes following rehabilitation and at 12 months. Hence the illness perceptions of patients should be considered by health personnel during rheumatology rehabilitation and their improvement may contribute to better outcomes. The RD-IPQ is a brief instrument that can be easily administered as part of routine care at the start of rehabilitation.

The rehabilitation institutions involved in this study did not include specific interventions aimed at improving illness perceptions. Future research could consider the effectiveness of such interventions in terms of short- and long-term improvements in illness perceptions and how they relate to other outcomes including symptoms and other aspects of health. Other longitudinal studies of illness perceptions have shown similar findings including significant changes in illness perceptions [29,30], however few studies have evaluated specific interventions that are designed to improve illness perceptions [13].

To our knowledge, this is one of the first studies to use an illness perceptions instrument that includes a summated rating that comprises important aspects of illness perceptions. The RD-IPQ has evidence for its psychometric properties in patients with rheumatic diseases [16] and includes illness perceptions of identity, consequences and emotions. Several studies have shown that these three aspects of illness perceptions are important in relation to health and outcomes [26,31]. However other aspects of illness perceptions that it does not include, may also be important in explaining variations in outcome for these patients. The theory proposes that different aspects of illness perceptions may contribute to different outcomes [9].

Other potential study limitations include the lack of information on non-respondents. The recruitment methods meant that information was not available to assess response bias at baseline. The loss to follow-up of approximately 30% at 12 months following one reminder is some cause for concern but there were no statistically significant differences in the baseline characteristics of the respondents and non-respondents to the follow-up questionnaire. The use of additional reminders might have improved the response rates at follow-up.

Automated selection procedures in regression including the forward stepwise method used here, have been criticised but can have a useful explorative purpose [23]. In particular, when two or more variables are highly correlated it can be a matter of chance as to which enters the equation. Several scales from the SF-36 which assessed aspects of health similar to those of the MHAQ and NRS scales were not included and the results of testing for multicollinearity were satisfactory. The statistical methods used are only appropriate for testing for association and hence no conclusions can be drawn causality and the relationship between illness perceptions and health outcomes. Future studies should consider using structural equation modelling (SEM) to further assess the relationship between illness perceptions, health and outcomes.

## Conclusion

Evidence was found for an association between illness perceptions, health and health outcomes as assessed by widely used instruments within rheumatology. Future studies should seek to verify these findings in other groups of patients with rheumatic disease and consider the role of rehabilitation in improving illness perceptions as a means of enhancing other health outcomes.

## Abbreviations

MHAQ: Modified Health Assessment Questionnaire; NRRK: National resource centre for rehabilitation in rheumatology; NRS: Numerical Rating Scale; RD-IPQ: The Rheumatic Disease Illness Perception Questionnaire; SEM: Structural Equation Modelling; SF-36: Short -Form 36- item (SF-36) health survey; WHO: The World Health Organisation.

## Competing interests

The authors declare that they have no competing interests.

## Authors’ contributions

IL contributed in the data collection, the statistical analysis and drafted the manuscript. EF revised the manuscript critically. AMG participated in the statistical analysis and revised the manuscript critically. All authors contributed in the design of the study and read and approved the final manuscript.

## Pre-publication history

The pre-publication history for this paper can be accessed here:

http://www.biomedcentral.com/1471-2474/14/28/prepub
